# Clearance of an immunosuppressive virus from the CNS coincides with immune reanimation and diversification

**DOI:** 10.1186/1743-422X-4-53

**Published:** 2007-06-06

**Authors:** Henning Lauterbach, Phi Truong, Dorian B McGavern

**Affiliations:** 1 Molecular and Integrative Neurosciences Department, The Scripps Research Institute, 10550 North Torrey Pines Rd., La Jolla, CA 92037, USA; 2 Harold L. Dorris Neurological Research Institute, The Scripps Research Institute, 10550 North Torrey Pines Rd., La Jolla, CA 92037, USA

## Abstract

Once a virus infection establishes persistence in the central nervous system (CNS), it is especially difficult to eliminate from this specialized compartment. Therefore, it is of the utmost importance to fully understand scenarios during which a persisting virus is ultimately purged from the CNS by the adaptive immune system. Such a scenario can be found following infection of adult mice with an immunosuppressive variant of lymphocytic choriomeningitis virus (LCMV) referred to as clone 13. In this study we demonstrate that following intravenous inoculation, clone 13 rapidly infected peripheral tissues within one week, but more slowly inundated the entire brain parenchyma over the course of a month. During the establishment of persistence, we observed that genetically tagged LCMV-specific cytotoxic T lymphocytes (CTL) progressively lost function; however, the severity of this loss in the CNS was never as substantial as that observed in the periphery. One of the most impressive features of this model system is that the peripheral T cell response eventually regains functionality at ~60–80 days post-infection, and this was associated with a rapid decline in virus from the periphery. Coincident with this "reanimation phase" was a massive influx of CD4 T and B cells into the CNS and a dramatic reduction in viral distribution. In fact, olfactory bulb neurons served as the last refuge for the persisting virus, which was ultimately purged from the CNS within 200 days post-infection. These data indicate that a functionally revived immune response can prevail over a virus that establishes widespread presence both in the periphery and brain parenchyma, and that therapeutic enhancement of an existing response could serve as an effective means to thwart long term CNS persistence.

## Background

Viral infections of the central nervous system (CNS) can remain asymptomatic or result in long-lasting neurological dysfunction, and in some extreme cases, death. Viruses that infect the CNS include herpesviruses, rhabdoviruses, retroviruses, picornaviruses, flaviviruses and arenaviruses (reviewed in [1]). Upon entry the means by which viruses adversely affect the CNS consist of direct mechanisms such as cellular lysis and blockade of cellular function or indirect mechanisms mediated by infiltrating immune cells attempting to ward off the invading pathogen. In fact, under certain conditions, the immune response necessary to eliminate the infectious agent can actually become detrimental to the host [2-4]. To limit the degree of immunopathology within the CNS, strong evolutionary pressures have likely led to the acquisition of several immune dampening mechanisms, such as compartmentalization behind a non-fenestrated blood-brain-barrier (BBB) and the limited expression of antigen-presenting machinery (i.e., major histocompatibility complex class I and II) (reviewed in [5,6]). The downside of this tight immune regulation is that a multitude of pathogens can exploit this weakness in order to establish long term persistence in CNS resident cells. Because the CNS is fraught with mechanisms to limit the toxicity (and most likely the effectiveness) of the immune response, it is surmised that this tissue compartment provides a favorable environment for prolonged viral persistence and neurologic dysfunction long after sterilizing immunity is achieved in the periphery (i.e., the route through which neurotropic viruses enter naturally).

Fetal infection in humans with lymphocytic choriomeningitis virus (LCMV) can lead to serious neurological complications, such as microcephaly, hydrocephalus, reduced mitosis in developing brain cells and mental retardation [7]. If mice are infected at birth or *in utero *with LCMV, neurons are the predominant cell population in the CNS parenchyma that harbor the virus [8]. Intravenous infection of adult mice with the parental strain of LCMV referred to as Armstrong results in an acute infection, which is resolved by virus-specific CD8 and CD4 T cells within 8–10 days [9]. In contrast, viral variants have been isolated that abort the T cell response and establish persistence in multiple tissues [10-16]. The prototypic member of this viral family is referred to as clone 13 and differs from wild type LCMV Armstrong by only two amino acids [10-12,14]. Clone 13 infection shares some of the features associated with persistent HIV-1 infection in humans, including infection/impairment of dendritic cells (DC) [15], exhaustion/deletion of the virus-specific T cell response [17-21], and the rapid establishment of viral persistence in the CNS as well as the periphery [20]. Interestingly, despite immune exhaustion (i.e., functional hyporesponsiveness of T cells), the virus-specific immune response eventually reacquires effector function and is able to clear clone 13 from peripheral tissues such as the blood, spleen, and liver [15,20]. However, studies have shown that clone 13 continues to persist in the CNS past the time when the virus is purged in the periphery [20].

Presently, it is not known why clone 13 continues to persist in the CNS for an extended time frame following viral clearance from the periphery [20], nor is it known which cell population(s) residing in the brain parenchyma harbors clone 13 during the early and late phases of persistence. It is also not known which elements of the cellular immune response enter the CNS in response to clone 13. In this study we set out to address these unanswered questions by simultaneously analyzing clone 13 tropism as well as the responding anti-viral immune response within the CNS. We demonstrate that clone 13 completely inundated the brain parenchyma with delayed kinetics when compared to peripheral tissues. Within the CNS parenchyma clone 13 sought early refuge within astrocytes and later infected olfactory bulb neurons before it was eventually purged from the entire compartment. When the functionality of the infiltrating CTL response was examined over this protracted clearance phase, signs of CTL exhaustion were evident but never as severe as that observed in peripheral tissues such as the spleen and liver. Interestingly, during the "functional reanimation" phase, a time period when the anti-viral CTL response regained functionality in all tissues, a major shift in the composition of the CNS immune repertoire was observed. Most notably, CD4 T and B cells increased both in frequency and cell number within the CNS during this phase. This coincided with a dramatic reduction in the number of persistently infected astrocytes and the eventual eradication of clone 13 from the CNS. These data provide a framework for understanding the cellular constituents responsible for purging an established persistent infection from the CNS and should facilitate future studies that aim to identify the precise mechanism(s) of clearance.

## Methods

### Mice

C57BL/6 (*H*-2^*b*^, Thy1.2^+^) and C57BL/6 Thy1.1^+^D^b^GP_33–41 _TCR-tg (P14) mice were bred and maintained in a closed breeding facility at The Scripps Research Institute. The handling of all mice conformed to the requirements of the National Institutes of Health and The Scripps Research Institute animal research committee.

### Virus

Six- to eight-week-old C57BL/6 mice were infected intravenously (i.v.) with 2 × 10^6 ^PFU of LCMV Armstrong clone 53b or LCMV Clone 13 to generate acute or persistent infection, respectively. Stocks were prepared by a single passage on BHK-21 cells, and viral titers were determined by plaque formation on Vero cells. The phenotypic and genotypic characterization of both LCMV strains, their passage, and viral plaque assays for quantification are described elsewhere [22].

### RT-PCR and Mnl I digestion from CNS viral clones

The RT-PCR and *Mnl *I digestion procedures were performed as described [13]. Briefly, brain homogenate was subjected to a standard plaque assay. Single plaques were picked and transferred into individual wells with a monolayer of BHK-21 cells. After two days total RNA was isolated (TRI REAGENT, Molecular Research Center, Inc.) and transcribed into cDNA using SuperScript III Reverse Transcriptase and random hexamer primers (Invitrogen). PCR was performed on the cDNA product with primers specific for the LCMV GP resulting in a 362 bp long DNA fragment. 10 μg of the PCR product were digested with *Mnl *I (NEB) and analyzed by agarose gel electrophoresis. This method allows detection of the U-to-C change at nucleotide 855 in the viral RNA of clone 13, which creates a cleavage site for *Mnl *I.

### T cell isolation and adoptive transfers

CD8 T cells were purified from the spleens of naïve P14 mice by negative selection (StemCell Technologies), and 5 × 10^3 ^purified cells were transferred i.v. into C57BL/6 mice. The mice were then infected 1–2 days later with LCMV.

### Mononuclear cell isolations and tissue processing

To obtain cell suspensions for flow cytometric analyses and stimulation cultures, the spleens, livers and CNS were harvested from mice after an intracardiac perfusion with a 0.9% saline solution to remove the contaminating blood lymphocytes. If noted, organs were incubated with 1 ml collagenase D (1 mg/ml; Roche) at 37°C for 20 min. Single-cell suspensions were then prepared by mechanically disrupting the organs through a 100-μm filter. Spleen cells were treated with red blood cell lysis buffer (0.14 M NH_4_Cl and 0.017 M Tris-HCl, pH 7.2), washed twice, and analyzed. Intrahepatic lymphocytes were further isolated by centrifugation in 35% Percoll (Amersham Biosciences) and then subjected to red blood cell lysis. To extract brain-infiltrating leukocytes, homogenates were resuspended in 90% Percoll (4 ml), which was overlaid with 60% Percoll (3 ml), 40% Percoll (4 ml), and finally 1× HBSS (3 ml). The Percoll gradients were then centrifuged at 1,500 rpm for 15 min, after which the band corresponding to mononuclear cells was carefully extracted, washed, and, ultimately, analyzed. The number of mononuclear cells was determined from each organ preparation and used to calculate the absolute number of specific cell populations. For immunohistochemical analyses, fresh, unfixed tissues were frozen on dry ice in optimal cutting temperature (OCT; Tissue-Tek). For the detection of infectious virus in the CNS, brains were cut sagittally and then half was homogenized using a Mini Beadbeater (BioSpec Products). Homogenates were analyzed using a standard plaque assay on Vero cells.

### Flow cytometry and intracellular cytokine staining

The following antibodies purchased from BD Biosciences were used to stain splenocytes as well as intrahepatic and brain-infiltrating leukocytes: anti-CD3-PE, anti-CD4-APC-Cy7, anti-CD11b-PE-Cy7, anti-CD11c-APC, CD19-PerCP-Cy5.5, anti-CD45.2-FITC, anti-NK1.1-PE, anti-Thy1.1-PerCP, anti-Thy1.2-PE, anti-TNFa-FITC, anti-IFNγ-PE and anti-IL-2-APC. Anti-CD8-Pacific Blue was purchased from Caltag. Before staining, all cell preparations were blocked with 3.3 μg/ml anti-mouse CD16/CD32 (Fc block; BD Biosciences) in PBS containing 1% FBS for 10 min. The Fc block was also included in all 20 min surface stains. For intracellular cytokine staining cell suspensions were stimulated for 5 hrs with 5 μg/ml of a dominant CD8 epitope mapping to amino acids 33–41 of the LCMV glycoprotein (GP_33–41_) in the presence of 50 U/ml recombinant IL-2 (NIH) and 1 μg/ml brefeldin A (Sigma). Afterward, cells surface stained with CD8-Pacific Blue and Thy1.1-PerCP and were then simultaneously fixed/permeabilized with a paraformaldehyde-saponin solution and, finally, stained with antibodies directed against IFN-γ, TNF-α and IL-2. Cells were acquired using a digital flow cytometer (Digital LSR II; Becton Dickinson) that allows up to 10-color detection by using four different excitation lasers. Flow cytometric data were analyzed with FlowJo software (Tree Star, Inc.). Gates for cytokine analyses were set based on non-peptide-stimulated controls and cells that stained negative for the protein of interest.

### Immunohistochemistry

**Figure 2:**
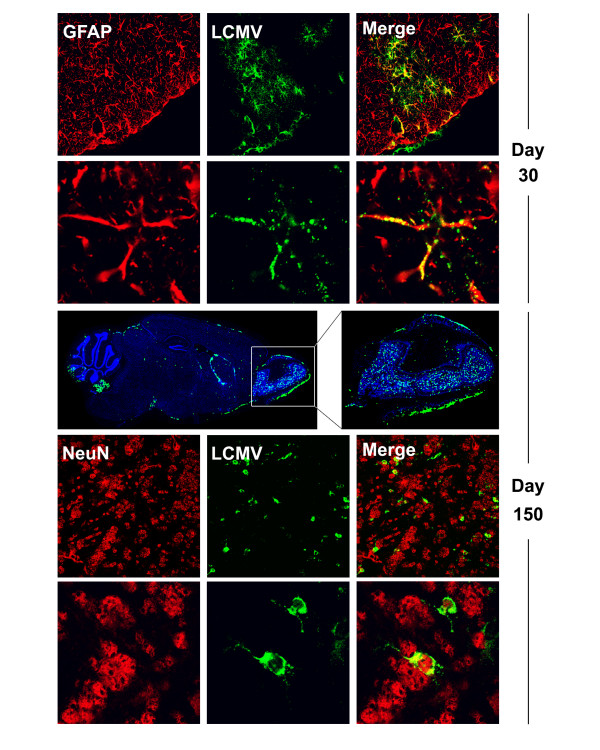
**Clone 13 tropism in the brain parenchyma during persistence**. The localization of clone 13 in the brain parenchyma was examined at various time points post-infection by two-color confocal microscopy. During the first 60 days the virus (green) was found primarily in GFAP^+ ^astrocytes (red). Representative low (first row) and high (second row) magnification images are shown for a mouse (n = 3 mice per group) at day 31 p.i. The third row shows a whole brain reconstruction from a mouse (n = 3 mice per group) at day 150 and an enlarged panel of the olfactory bulb. Virus is shown in green and cell nuclei in blue. In the late phase of persistence (day 150), the virus (green) was found primarily in NeuN^+ ^olfactory bulb neurons (red). Low and high magnification examples are shown in the fourth and fifth rows, respectively.

To visualize LCMV, astrocytes, and neurons, 6-μm frozen sections were cut, fixed with 2% formaldehyde, blocked with an avidin/biotin-blocking kit (Vector Laboratories), and stained for 1 h at room temperature with guinea pig anti-LCMV (1:1500), rabbit anti-glial fibrillary acidic protein (anti-GFAP; 1:800; DakoCytomation), or 1.25 μg/ml of mouse anti-neuronal nuclei (anti-NeuN; Chemicon International), respectively. To block endogenous mouse antibodies, sections stained with mouse anti-NeuN were pre-incubated for 1 hr at room temperature with 35 μg/ml of a Fab anti-mouse H and L chain antibody (Jackson ImmunoResearch Laboratories). After the primary antibody incubation, sections were washed, stained for 1 h at room temperature with a biotinylated secondary antibody (1:400; Jackson ImmunoResearch Laboratories), washed, and stained for 1 h at room temperature with streptavidin-Rhodamine Red-X (1:400; Jackson ImmunoResearch Laboratories). For co-labeling of LCMV and NeuN or LCMV and GFAP (Fig. 2), frozen sections were stained as described above except that the anti-LCMV antibody was detected with an anti-guinea pig secondary antibody directly conjugated to FITC (1:750; for 1 h at room temperature). All sections were co-stained for 5 min at room temperature with 1 μg/ml DAPI (Sigma-Aldrich) to visualize cell nuclei. All working stocks of primary and secondary reagents were diluted in PBS containing 2% FBS.

### Microscopy

**Figure 1:**
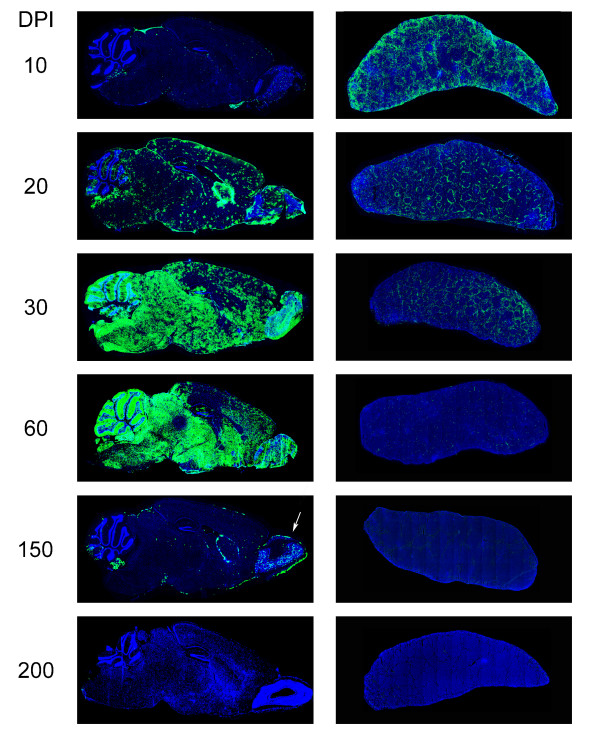
**Distribution of LCMV in the brain and spleen following an intravenous clone 13 infection**. Representative sagittal brain and spleen reconstructions (n = 3 mice per group) were assembled at the denoted time points post-infection to reveal the distribution of LCMV (green) following an intravenous infection with 2 × 10^6 ^PFU of clone 13. Note the minimal amount of virus in the brain at day 10 and the complete inundation of the brain parenchyma by day 30. During the late phase of persistence (day 150), clone 13 localizes primarily to the olfactory bulb (white arrow) and also maintains a presence in the meninges, choroid plexus, ependyma, and subventricular zone. Note that the spleen shows the highest viral antigen load at day 10 and is progressively purged of virus over time. Cell nuclei are shown in blue.

Two-color organ reconstructions (Fig. 1) to visualize the distribution of LCMV on 6-μm frozen sections were obtained using an immunofluorescence microscope (Axiovert S100; Carl Zeiss MicroImaging, Inc.) fitted with an automated xy stage, a color digital camera (Axiocam, Carl Zeiss MicroImaging, Inc.), and a 5× objective. Registered images were captured for each field on the tissue section, and reconstructions were performed using the MosaiX function in KS300 image analysis software (Carl Zeiss MicroImaging, Inc.). Higher resolution images of LCMV-infected neurons or astrocytes (Fig. 2) were captured with a confocal microscope (MRC1024; Bio-Rad Laboratories) fitted with a krypton/argon mixed gas laser (excitation at 488, 568, and 647 nm) and a 40× oil objective (Carl Zeiss MicroImaging, Inc.). All two-dimensional confocal images illustrate a single z section captured at a position approximating the midline of the cell.

### Statistical analyses

Data handling, analysis, and graphical representations were performed using Microsoft Excel 2003 and SigmaPlot 9.0 (Systat). Statistical differences were determined by Student's *t *test or Mann-Whitney Rank Sum Test (P < 0.05) using SigmaStat 3.1 (SigmaStat).

## Results

### CNS viral clearance is delayed in mice infected intravenously with LCMV clone 13

High dose infection of adult mice with LCMV clone 13 results in a chronic viral infection during which the virus distributes systemically both in lymphoid and non-lymphoid tissues [12,20,23,24]. In nearly all peripheral tissues, clone 13 is purged within 2 to 3 months [20]; however, a few studies have suggested that virus might persist for the lifetime of the host in the CNS [11,20]. This is of particular interest because the CNS is an immunologically specialized compartment [6,25] known to limit the effectiveness of adaptive immune response. Thus, it is plausible that once a virus like clone 13 establishes long term persistence within the CNS it is difficult (if not impossible) to completely remove.

In order to obtain a detailed understanding of clone 13 distribution kinetics and tropism within the CNS, we infected adult C57BL/6 mice intravenously with 2 × 10^6 ^PFU clone 13 and then monitored viral spread in spleen, liver and brain by immunohistochemistry (Fig. 1). In contrast to the spleen (Fig. 1) and the liver (data not shown), where antigenic load peaked at day 10 post infection (p.i.), the brain parenchyma was not fully inundated with clone 13 until day 30 (Fig. 1). Titers of infectious virus in the CNS as measured by plaque assay reached their maximum level by day 20 p.i., and this titer was maintained until day 60, at which point a steady decline in viral titers was noted both by plaque assay (Table 1) as well as immunohistochemistry (Fig. 1).

**Table 1:** Brain Viral Titers. Kinetics of viral clearance from the brain. Clone 13 infected mice were perfused with saline and then brains were isolated at the denoted days post infection (DPI). The titer of infectious virus was determined by plaque assay and is expressed as plaque forming units (PFU) per gram tissue. The lower limit of detection is 200 PFU/g of tissue

**DPI**	**Brain Virus Titer (PFU/g)**
10	4.04 × 10^5^
20	3.90 × 10^6^
30	2.16 × 10^6^
60	1.16 × 10^5^
150	5.73 × 10^3^
200	< 200

Interestingly, and in support of previous studies [20], the pattern of clearance in the CNS did not closely mirror that of peripheral tissues such as the spleen and liver. Whereas the blood (data not shown), liver (data not shown), and spleen (Fig. 1) were completely purged of virus within 60–80 days of infection, CNS virus was not finally resolved until around day 200 (Fig. 1, Table 1). However, coincident with the clearance of clone 13 from the periphery around day 60 was a marked shift in the distribution of virus within the brain parenchyma. Between day 60 and 150, clone 13 was purged to a large degree from the brain parenchyma. In fact, the choroid plexus, meninges, subventricular zone, and, most notably, the olfactory bulb, served as the last bastions of virus (see day 150, Fig. 1) before the pathogen was finally purged at day 200 (Fig. 1). These data demonstrate that despite the establishment of long term persistence within the CNS, clone 13 can ultimately be eliminated from this compartment; however, the kinetics of clearance differ significantly from most peripheral tissues.

### Pattern and tropism of LCMV clone 13 in the CNS

Because the virus was introduced into the blood supply, it is no surprise that brain infection was initiated around blood vessels at early time points post-infection. This gave rise to a punctate pattern of viral antigen staining on sagittal brain reconstructions at day 10 p.i. (Fig. 1). At these early time points, clone 13 antigen could also be found in choroid plexus, meninges, and ependymal cells – the traditional targets of LCMV introduced intracerebrally [26]. From the vascular seeds, it is likely that the astrocyte, whose foot processes line the blood brain barrier, served as the portal of clone 13 entry into the brain parenchyma. When the tropism of the virus was examined at one month post-infection by co-staining for LCMV and GFAP (astrocytes) or NeuN (neurons), it was revealed that all of the parenchymal LCMV staining overlapped with GFAP (Fig. 2) not NeuN (data not shown), supporting the notion that astrocytes are the preferred parenchymal target for clone 13 introduced intravenously. By day 20 post-infection, clusters of antigen could be observed throughout the parenchyma (Fig. 1), and the virus appeared to be moving from cell-to-cell (Fig. 2). This finally progressed to near complete inundation of the parenchyma at day 30 p.i. – a state that remained until day 60. Interestingly, during this progression the corpus callosum and neocortex were never infected to the same degree as the remainder of the brain parenchyma.

Following day 60 a dramatic change in the distribution of clone 13 was noted in the CNS parenchyma. By day 150 p.i., a time point when spleen was completely purged of clone 13, viral antigen was substantially reduced in the brain parenchyma, but could still be found in the choroid plexus, meninges, subventricular zone, and olfactory bulb (Fig. 1). Interestingly, at this late phase of persistence, clone 13 appeared to have acquired a new target. Co-staining analyses revealed that in addition to ependymal cells, meningeal cells, and cells comprising the choroid plexus, clone 13 had spread to olfactory bulb neurons (Fig. 2). These data demonstrate that for the first two months of persistence, clone 13 primarily infects astrocytes within the brain parenchyma, but establishes late phase persistence in olfactory bulb neurons before it is finally cleared at day 200 post-infection.

### Neurotropic Armstrong is not selected for over time in the CNS of clone 13 infected mice

**Figure 3:**
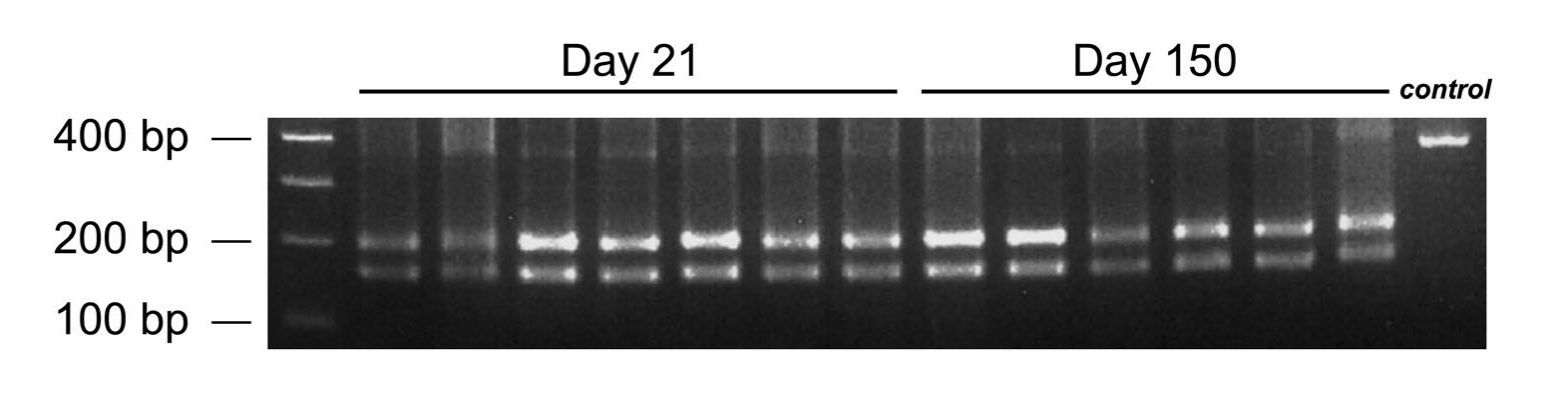
**Neurotropic LCMV Armstrong is not selected for in the CNS of clone 13 infected mice**. RNA was isolated from LCMV clones extracted from the brains of mice at 21 (n = 7 clones) and 150 days (n = 6 clones) post-clone 13 infection. RT-PCR, PCR and *Mnl *I restriction enzyme digests were performed as described in the Materials and Methods. The RNA PCR product from the Armstrong GP contains a phenylalanine at position 260 and is not cleaved by *Mnl *I. In contrast, clone 13 contains a leucine at position 260, and the 362 bp PCR product is cleaved into fragments (202 and 160 bp) by *Mnl *I. Note that all clones analyzed at both time points had the *Mnl *I restriction enzyme site. The control lane shows undigested 362 bp GP PCR product.

The localization of LCMV in olfactory bulb neurons during the late phase of persistence suggested that the CNS selected for the more neurotropic strain of LCMV (i.e., Armstrong) over time. There is precedence in the literature to support that Armstrong can out-compete clone 13 when both are simultaneously administered into the CNS [27]. Moreover, examination of viral clones extracted from the CNS of LCMV carrier mice persistently infected from birth has revealed that Armstrong is usually found in the CNS and clone 13 in peripheral lymphoid tissues [11]. To determine if Armstrong was selected for in the CNS of clone 13 infected mice over time, we examined viral clones of LCMV extracted from the CNS at an early (day 8) versus a late time point (day 150) p.i. The glycoprotein of each clone was amplified by RT-PCR and then subjected to a *Mnl *I restriction digest. It was demonstrated previously that this assay provides a simple means to detect the U-to-C change at nucleotide 855 in the viral RNA of clone 13 [13]. Our results revealed that 100% of the clones analyzed at both time points retained the *Mnl *I restriction site (Fig. 3). Therefore, the neurotropic Armstrong strain of LCMV was not selected for over time in the CNS.

### Dynamics of LCMV specific CTL responses

The delayed clearance kinetics in the CNS compared to periphery (e.g., the spleen and liver) led us to examine the LCMV-specific CD8 T cell response during both the acute and chronic phases of persistence. As a positive control for these studies, we simultaneously examined the CTL response to LCMV Armstrong, which following intravenous inoculation is readily cleared from all tissues within 10 days. Because LCMV clone 13 differs by only two amino acids from the parental Armstrong strain [22,24], all known T cell epitopes are preserved, rendering these two viruses particularly amenable to study. In order to monitor the generation and maintenance of virus-specific CTL over time, we opted to study a traceable population of LCMV-specific T cell receptor (TCR) transgenic (tg) cells specific to amino acids 33–41 of the LCMV glycoprotein (GP) (D^b^GP_33–41_) [28]. These cells have been used routinely in the field to provide a traceable representative of the endogenous CTL response [29-31]. The advantage of using TCR-tg cells is that the fate of a single LCMV-specific T cell population with a known TCR can be followed from the initial infection to the late phase of persistence without the contaminating influence of new thymic emigrants that emerge throughout infection [32].

**Figure 4:**
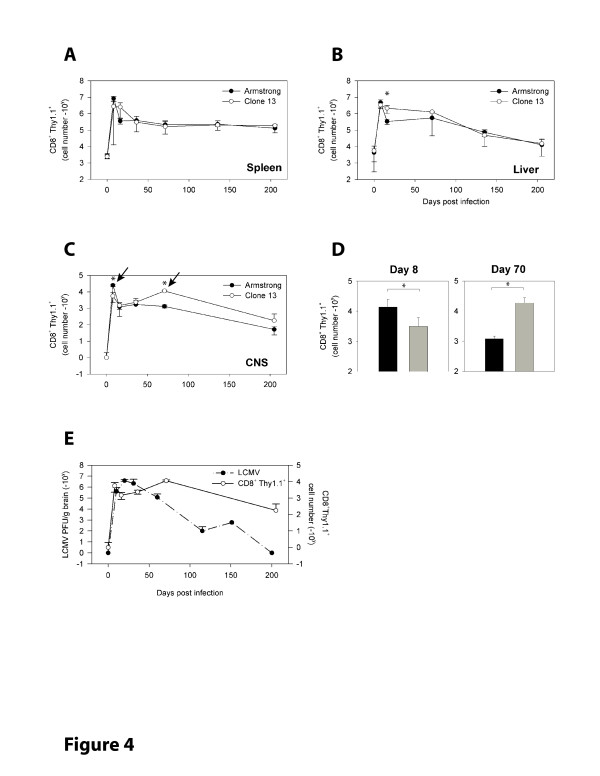
**Kinetics of the LCMV specific CD8 T cell response**. Mice were seeded with Thy1.1^+^D^b^GP_33–41 _specific CD8^+ ^T cells (P14 cells) and infected one day later with 2 × 10^6 ^PFU of Armstrong or clone 13. Mononuclear cells were isolated from the **A) **spleen, **B) **liver and **C) **CNS at the indicated time points following intracardiac perfusion to remove contaminating blood cells. The absolute number of P14 cells in each tissue was determined by flow cytometry. A log fewer CTL was found in the CNS of clone 13 infected mice at day 8 p.i. (first black arrow), and a significant elevation in P14 cells was observed at day 71 (second black arrow). Values represent the mean ± standard deviation (SD) of three mice per group at each time point. No significant differences were noted in the spleen or liver (two representative peripheral tissues). **D) **To confirm the findings in panel C, CNS P14 cells were quantified in a separate experiment (n = 4 to 7 mice per group) at an early (day 8) and late (day 70) time point post-infection. Note the significant reduction in P14 cells at day 8 and the elevation at day 70 when clone 13 infected (gray bars) were compared to Armstrong infected (black bars) mice. Data are represented as the mean ± SD. Asterisks denote statistically significant (*p *< 0.05) differences between Armstrong and clone 13 infected mice. **E) **The absolute number of CD8^+^Thy1.1^+ ^P14 cells (open circles) in the CNS of clone 13 infected animals (as shown in panel C) is plotted against the titer of infectious virus (black circles) in the brain at various time points after clone 13 infection (as shown in Table 1). Note that the elevation in CNS CTL numbers coincides with a reduction in infectious virus as determined by plaque assay.

To approximate the physiological number of endogenous precursors [33], we adoptively transferred 5 × 10^3 ^naïve Thy1.1^+ ^D^b^GP_33–41 _specific TCR-tg CD8^+ ^T cells (referred to as P14 cells) into Thy1.2^+ ^C57BL/6 mice 1–2 days before infection with 2 × 10^6 ^PFU of LCMV Armstrong or clone 13. Following infection with Armstrong or clone 13, P14 cells initially expanded with similar kinetics in the spleen, liver and CNS, although the magnitude of the response was reduced in clone 13 infected hosts, especially within the CNS (Fig. 4C). Within the CNS a statistically significant (*p *= 0.002) 4-fold reduction in the absolute number of P14 cells was observed at day 8 p.i. (Fig. 4C,D). The marginal differences noted in the spleen and liver did not reach statistical significance. During the contraction phase following day 10 p.i., P14 cell numbers remained elevated in the spleen and liver of clone 13 infected mice, but were eventually reduced to a steady state level comparable to that observed in Armstrong infected mice within one month of infection (Fig. 4A,B). This steady state level was then maintained for the entire examination period (200 days). Interestingly, at around day 70 post-infection, a statistically significant (*p = *0.016) 16-fold increase in the absolute number of P14 cells was observed in the CNS (Fig. 4C,D), but not the spleen or liver (Fig. 4A,B) of clone 13 infected mice when compared to Armstrong. This increase coincided temporarily with the decline in virus observed by both plaque assay (Table 1 and Fig. 4E) and immunohistochemistry (Fig. 1). It is also worth noting that P14 cells were maintained in the CNS of Armstrong infected mice for the entire observation period despite our inability to detect virus at any time point following day 10, supporting the notion that memory CTL are maintained in the CNS in the absence of antigen [34,35]. Nevertheless, the marked increase of P14 cells observed in clone 13 infected mice suggests an antigen-driven process.

### Differential preservation of CTL function in clone 13 infected mice

**Figure 5:**
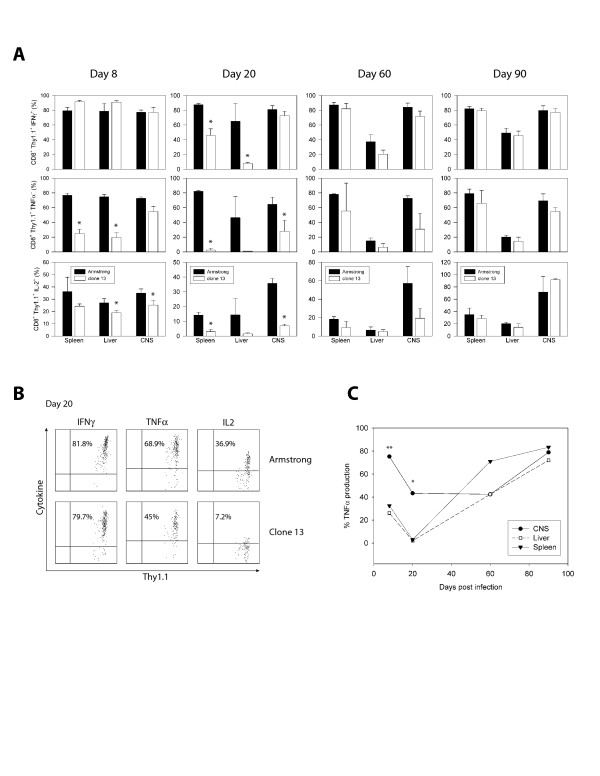
**Analysis of CTL function during clone 13 persistence**. **A) **Mononuclear cells were extracted from the spleen, liver and CNS of Armstrong (black bars) or clone 13 (white bars) infected mice (n = 3 mice per group) at the denoted time points. Following a 5 hr *in vitro *stimulation with GP_33–41 _peptide, P14 cells were examined flow cytometrically for the production of IFN-γ (top row), TNF-α (middle row) and IL-2 (lower row). Note that when compared to the P14 cells in the spleen and liver, an intermediate state of P14 functional exhaustion was observed in the CNS. This was most prominent at day 20 p.i. P14 cells in all compartments regained complete functionality by day 90 p.i. Each bar represents the mean ± SD. Statistical differences between Armstrong and clone 13 infected mice are denoted by asterisks (*p *< 0.05). **B) **Representative dot plots used to generate the bar graphs in panel A are shown for CNS P14 cytokine production at day 20 p.i. This time point was selected to show the relative preservation of CNS P14 function at a time point when functional exhaustion was most severe in the spleen and liver. Dot plots are gated on CD45^+^CD8^+^Thy1.1^+ ^P14 cells, and the numbers indicate the frequency of P14 cells that produce the denoted cytokines. **C) **The relative loss in P14 function was calculated by dividing the frequency of TNF-α producing P14 cells (as shown in panel A) from the CNS, spleen, and liver of clone 13 infected mice by the frequency observed in Armstrong infected mice. This number was multiplied by 100 to generate percentages. Note the relative preservation of P14 function in the CNS when compared to peripheral tissues. Double asterisks (**) denote a statistically significant difference (*p *< 0.05) between the CNS and spleen as well as the CNS and liver. A single asterisk (*) denotes a statistically significant difference (*p *< 0.05) between the CNS and spleen only.

One hallmark of chronic infection with clone 13 is the gradual functional impairment of LCMV specific CD8^+ ^and CD4^+ ^T cells [17,20,21,36] – a phenomenon referred to as immune exhaustion [17]. The functional impairment is characterized by a progressive loss in the capacity of T cells to produce cytokines such as IL-2, TNF-α and IFN-γ upon antigenic stimulation. Given the unique pattern of viral clearance within the CNS of clone 13 infected mice, we set out to analyze the functional state of LCMV-specific CTL in the CNS versus the periphery. Evidence of functional exhaustion was readily apparent in the spleen and liver within 8 days of clone 13 infection (Fig. 5A, day 8). This was evidenced by statistically significant reduction in the ability of P14 cells to produce IL-2 and TNF-α. At this time the ability of CNS-derived P14 cells to produce IL-2 and TNF-α also started to wane, but to a much lesser degree than observed in the peripheral tissues (Fig. 5A,C). Immune exhaustion in P14 cells peaked at day 20 post-clone 13 infection, a time point when P14 cells in spleen and liver had almost no ability to produce IL-2 and TNF-α, and a statistically significant reduction in IFN-γ production was also observed (Fig. 5A, day 20). CNS-derived P14 cells also showed some evidence of functional exhaustion at this time, but again to a lesser degree that observed in the periphery (Fig. 5A,C). Approximately 7% of CNS P14 cells produced IL-2 (compared to 3.0% in the spleen and 1.5% in the liver) and ~28% produced TNF-α (compared to 2.5% and 0.9% in spleen and liver, respectively) (Fig. 5A,B). In addition, no significant reduction in IFN-γ-producing P14 cells was observed in the CNS. By day 60 post-infection P14 cells started to regain the ability to produce cytokines in response to antigen (Fig. 5A, day 60), and by day 90 P14 functionality was fully restored in all tissues examined (Fig. 5A, day 90). These data show that in the clone 13 system, CTL exhaustion is followed by a period of "functional reanimation". In addition, the severity of CTL exhaustion in the CNS was never as great as that observed in peripheral tissues.

### The "functional reanimation" phase is associated with diversification in the CNS immune repertoire

The time course of sagittal brain reconstructions revealed that clone 13 established widespread infection of the brain parenchyma predominantly in astrocytes and that the virus was finally eliminated from this compartment following a transient state persistence in olfactory bulb neurons (Fig. 1, 2). We define the time period following day 60 as the "functional reanimation" phase because CTL cytokine-producing ability returns to normal levels both in the periphery and CNS. During this period, viral titers in serum (not shown), liver (not shown), and spleen (Fig. 1) are reduced to background levels, and CNS virus begins a steady descent that requires >100 additional days before complete clearance is achieved. We became particularly interested in this time period because the adaptive immune system, despite passing through a state of functional exhaustion, ultimately gains the upper hand in the clone 13 system and purges virus from the immunologically specialized CNS. Therefore, we next examined the immunological factors associated with CNS viral clearance. Our CTL functional data demonstrate quite clearly that immune exhaustion in the CNS was never as severe as that observed in the spleen and liver (Fig. 5C), and at the time point when functional reanimation begins (~ day 60), a significant increase in P14 number and a coinciding decrease in viral titers was noted (Fig. 4E).

**Figure 6:**
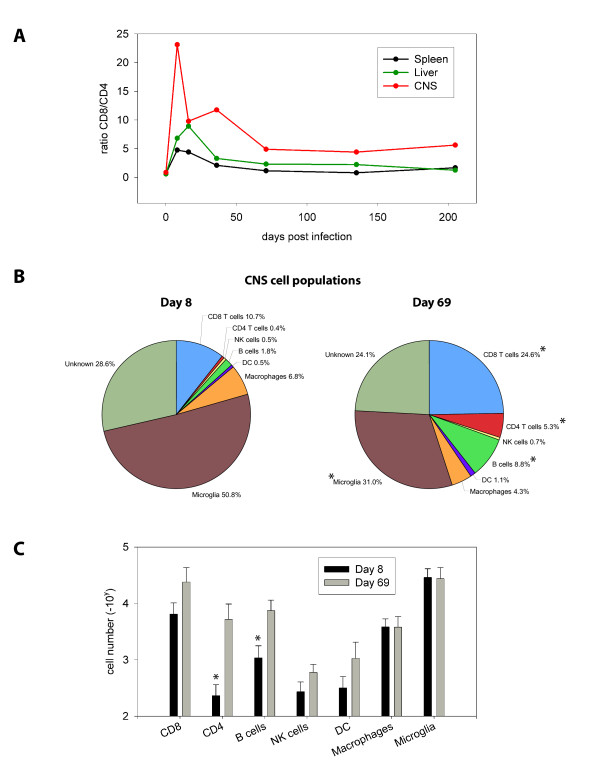
**Diversification in the CNS immune repertoire during the reanimation phase**. **A) **The CD8 to CD4 T cell ratio was calculated for spleen, liver, and CNS of clone 13 infected mice over time by dividing the absolute number of CD8 T cells extracted from each tissue by the absolute number of CD4 T cells. Note that the ratio is highest in the CNS at early time points post-infection. As the number of CD4 T cells increase in the CNS over time this ratio becomes similar to that observed in peripheral tissues. **B) **Mononuclear cells were extracted from spleen (data not shown), liver (data not shown) and CNS of clone 13 infected mice (n = 4 mice per group) at day 8 (exhaustion phase) or day 69 (reanimation phase) p.i. to define the immune repertoire. Multi-parameter digital flow cytometry permitted analysis of the entire immune repertoire from a single sample for each tissue. The frequencies of both innate and adaptive immune cells are represented as pie diagrams. Statistically significant increases (*p *< 0.05) at day 69 are denoted by asterisks. **C) **The frequencies shown in panel B were used to calculate the absolute number of the respective CNS cell populations in clone 13 infected mice. Note the statistically significant (*p *< 0.05) increase in the number of CD4 T cells and B cells in the CNS at day 69. The bars represent mean ± SD at each time point. Asterisks denote a statistically significant difference between day 8 and day 69.

However, because semi-functional CTL were maintained in the CNS throughout the immune exhaustion stage of infection, a time period when CNS viral loads were relatively high, we postulated that CTL alone might not be responsible for the eventual clearance of virus from the CNS. To address this possibility we quantified the cellular composition of CNS infiltrate during the reanimation phase in clone 13 infected mice not seeded with traceable P14 cells. When the ratio of bulk CD8 to CD4 T cells was calculated in the spleen, liver and CNS over a 200-day time window (Fig. 6A), we noted that, at the peak of the primary response (day 8 p.i.), the CNS-infiltrating T cell response was strongly dominated by CTL; there were 23 times more CD8 than CD4 T cells in the CNS on average. Interestingly, this CD8 dominance was unique to the CNS, because the spleen and liver at day 8 showed ratios of 4.7 and 6.8, respectively. Even after the contraction phase, the CNS still harbored 10 times more CD8 than CD4 T cells. However, at the start of the functional reanimation phase (day 60–70 p.i.), the ratio stabilized between 4–5 and remained there for the duration of the examination period.

The shift in the CD8:CD4 ratio during the reanimation phase prompted us to further investigate the entire immune repertoire in the CNS. For these complex analyses, we selected day 8 and 70 p.i. as time points representative of early functional exhaustion and reanimation, respectively. These were also the time points when we observed the most pronounced mononuclear infiltration into the CNS (Fig. 4), which facilitated analyses of CNS immune repertoires in individual mice. Using multi-parameter digital flow cytometry, we quantified seven distinct cell populations in single samples extracted from the CNS of individual mice: CD8 T cells (CD45^hi^Thy1.2^+^CD8^+^CD4^-^), CD4 T cells (CD45^hi^Thy1.2^+^CD4^+^CD8^-^), B cells (CD45^hi^NK1.1^-^Thy1.2^-^CD19^+^), NK cells (CD45^hi^CD4^-^CD8^-^CD11b^-^NK1.1^+^), dendritic cells (CD45^hi^NK1.1^-^Thy1.2^-^CD11c^+^), macrophages (CD45^hi^NK1.1^-^Thy1.2^-^CD11c^-^CD11b^+^), and microglia (CD45^low^NK1.1^-^Thy1.2^-^CD11b^+^). Most of the denoted cell populations were elevated both in frequency (Fig. 6B) and absolute number (Fig. 6C) at day 70 p.i. Only macrophages and microglia were found at lower frequencies (Fig. 6B), which did not affect absolute numbers (Fig. 6C). Dendritic cells and NK cells increased only slightly. The most dramatic changes occurred in the lymphocyte compartment, specifically CD4 T and B cells. The absolute number of CD4 T cell numbers increased more than 22-fold, and B cell numbers, 7-fold (Fig. 6C). Importantly, to demonstrate specificity in the bulk CD4 compartment at day 70 p.i., we utilized an I-A^b^GP_61–80 _MHC tetramer as described previously [21,37]. At day 70, 4.5% ± 2.6% of the CD4 T cells were specific for the immunodominant GP_61–80 _peptide presented in I-A^b^. CD8 T cells still represented the most predominant leukocyte population in the CNS at day 70, with their numbers increasing 4-fold from day 8. In addition, 11.7 ± 2.6% of the CD8 cells were determined to be GP_33–41 _specific by D^b^GP_33–41 _MHC tetramer staining. Importantly, all of the aforementioned changes were unique to the CNS at day 70 and not noted in the spleen or liver (data not shown). These data demonstrate collectively that at day 70 p.i., a time point when peripheral tissues are largely devoid of virus, the adaptive immune response in the CNS not only regains functionality (Fig. 5) but also diversifies its cellular repertoire (Fig. 6). This coincides with a decline in CNS viral titers (Fig. 4E).

## Discussion

The immunosuppressive variant of LCMV, clone 13, was first isolated over two decades ago from spleens of persistently infected carrier mice [12], and since that time infection with this isolate has provided a highly relevant paradigm to identify host factors that facilitate the establishment of systemic viral persistence [15,18,20,21,38,39]. Importantly, many of the lessons learned in the clone 13 system have direct correlates to immunosuppressive states induced during persistent infection of humans. As case in point is the recent identification of the PD-1 [38] and IL-10 pathways [39] as being involved in the immunosuppression observed during persistent infections of both mouse [38,39] and humans [40-42]. Another advantage of the clone 13 system that has not been exploited to any appreciable degree stems from the fact that the virus is purged almost entirely from the host over an extended time frame. Following a period of functional exhaustion [17,19], the adaptive immune system appears to reengage in clone 13 infected mice and purge virus systemically. The latter period, which we define as the "functional reanimation" phase, provides a desirable experimental paradigm, because the immune system, despite suffering through a state of immunosuppression, eventually achieves the upper hand without therapeutic intervention. Given that functional reanimation of the immune system is a coveted therapeutic aim in persistently infected humans, we propose that there is much to be learned by studying the natural progression and evolution of adaptive immunity in clone 13 infected mice. In the clone 13 system, nature provides instruction regarding how to control a systemically distributed persistent viral infection that simultaneously engages several immunosuppressive pathways [15,38,39].

At the outset of our studies, little was known about the progression of clone 13 infection within the CNS – a compartment of particular interest given its immunologically specialized status [6,25] and its unique susceptibility to irreparable consequences during viral persistence [1]. In this model it was known that most peripheral tissues were purged of clone 13 within 50 to 60 days, whereas the CNS remained replete with infectious virus at this time [43], suggesting the possibility that the adaptive immune system might not be equipped to cleanse the CNS of a persistent virus after progressing through an immunosuppressive state. Therefore, we initiated a series of studies to investigate the relationship between clone 13 and the adaptive immune response in the CNS over time. The results of these studies have led to four important findings that we believe advance our understanding of this model system. First, following introduction into the blood supply, we noted that clone 13 inundated the brain parenchyma more slowly than peripheral tissues, such as the spleen and liver. Second, within the brain parenchyma, we observed the clone 13 replicated initially in astrocytes and was later found in olfactory bulb neurons (one of the last bastions of viral persistence); however, despite this tropism shift, clone 13 was eventually purged from the CNS, albeit with delayed kinetics when compared to the periphery. Third, analyses of clone 13-specific CTL revealed their presence in the CNS early after infection, but their numbers were reduced when compared to an acute Armstrong infection. When CTL functionality was examined, we observed an intermediate state of functional impairment in anti-viral cytokine production during the exhaustion phase when clone 13 established a strong presence in the brain parenchyma. Fourth, during the reanimation phase, a time period when virus-specific CTL regained functionality and increased in number within the CNS, diversification of the CNS immune repertoire was observed, most notably an increase in the number of CD4^+ ^T cells and B lymphocytes. This diversification coincided with a dramatic reduction in the parenchymal virus load and the eventual eradication of the pathogen from the CNS over the ensuing months. These data suggest collectively that temporal diversification of the immune repertoire is nature's solution to the problem of removing immunosuppressive clone 13 from the murine CNS – a supposition that requires further experimentation to prove definitively.

To gain insights into clone 13 infection kinetics, we assembled temporal sequences of tissue reconstructions (periphery versus brain) to illustrate the expression of viral antigen over time. After an intravenous injection, clone 13 distributes systemically [12,23,24]. Following systemic distribution, the representative peripheral tissues we examined (i.e., spleen and liver) were fully inundated with virus by day 10, whereas complete infection of the brain parenchyma was not achieved until day 30 (Fig. 1). This delay is likely explained by the presence of a non-fenestrated blood-brain-barrier (BBB) in the CNS, which has an essential role in maintaining a highly regulated microenvironment for the proper neuronal functioning. The BBB is composed of astrocytic foot processes, endothelial cells, and their associated basement membranes [44]. Importantly, the receptor for LCMV clone 13, α-dystroglycan [45], is highly expressed on the astrocyte foot processes [46-48]. In fact, we propose that this explains the early targeting of astrocytes by clone 13. We also postulate that astrocytes likely serve as the portal for clone 13 entry into the CNS following intravenous inoculation. This is supported by our confocal analyses (Fig. 2) and the punctate pattern of viral antigen expression we observed around blood vessels on brain reconstructions at day 20 p.i. (Fig. 1). Astrocytes have also been described as an intermediary following neonatal infection of rats with the LCMV Armstrong-4 strain [49], and glial tumor cells can be transduced with LCMV-GP-pseudotyped lentiviral vectors [50]. Interestingly, the pattern of CNS infection in adult mice infected intravenously with clone 13 differs considerably from that observed following infection at birth or *in utero *[51,52]. When LCMV is injected into neonates, the resultant carrier mice reach adulthood with virus persisting solely in parenchymal neurons [52,53]. These adult carrier mice harbor the clone 13 variant of LCMV in the periphery [11], yet astrocytes remain devoid of virus. The precise variables that dictate the patterns of LCMV CNS tropism remain to be determined and are an active area of investigation within the laboratory.

Another interesting observation regarding clone 13 tropism relates to how the virus gains access to olfactory bulb neurons over time. We originally surmised that following an intravenous clone 13 infection, the CNS would select for the more neutropic Armstrong strain of LCMV, which differs from clone 13 by only two amino acids. Indeed, there is precedence in the literature to support that Armstrong can out compete clone 13 within the CNS [27], and reacquisition of the two amino acids required to revert clone 13 back to Armstrong was not inconceivable over the lengthy 6 month period of CNS persistence. However, examination of viral clones from the CNS at early and late time points revealed quite conclusively that clone 13 did not lose its *Mnl *I restriction enzyme site, evidence of conversion into the Armstrong strain [13,16]. While these data demonstrate that the Armstrong strain of LCMV was not generated, it remains possible that other variants of LCMV were selected for in the CNS of clone 13 infected mice. Sequence analyses of clones are required to address this possibility.

A second possibility to explain the transition to olfactory bulb neurons during the late phase of persistence could be the targeting of neural stem cells. Interestingly, lentiviral vectors pseudotyped with LCMV (WE54)-GP have been shown to transduce neural stem cells/progenitors *in vivo *[54]. Studies have demonstrated that GFAP-expressing type B astrocytes residing in the subventricular zone (SVZ) are the *in vivo *precursors of newly generated neurons in the adult mammalian brain [55]. Type B astrocytes give rise to rapidly dividing transit-amplifying cells, which further develop into migratory neuroblasts (reviewed in [56]). Forming tangential chains, these neuroblasts migrate along the rostral migratory stream (RMS) from the SVZ into the olfactory bulb, where they differentiate into two kinds of inhibitory neurons [57]. Given that lentiviral vectors pseudotyped with LCMV GP have been shown to target neural stem cells, it conceivable that clone 13 accesses olfactory bulb neurons in part through this pathway. Studies are underway to address this hypothesis.

Because clone 13 persisted in the CNS of mice for roughly 6 months before eradication, we considered analyses of the responding immune repertoire to be of great importance. We began by first examining a traceable representative of the virus-specific CTL response, namely P14 cells (or D^b^GP_33–41 _specific CTL). Approximately, 5-fold more P14 cells were recruited into the CNS of Armstrong versus clone 13 infected mice at day 8 p.i. Confirming results from previous studies [18,20], we also observed early evidence of CTL functional exhaustion around this time point. However, it should be noted that the severity of CTL exhaustion in the CNS of clone 13 infected mice was not as severe as that observed in peripheral tissues. Moreover, P14 cells in the CNS never lost the ability to produce the antiviral cytokine IFN-γ. The relative functional preservation of CTL in the CNS could be attributed to the low number of DCs in this compartment during the acute phase of clone 13 persistence (Fig. 7C). DCs in clone 13 infected mice were recently shown to be a major source of the immunosuppressive cytokine, IL-10, responsible for T cell inactivation and subsequent viral persistence in this model system [39]. A reduced number of CNS DCs could potentially expose CTL to a less immunosuppressive milieu.

An alternative possibility is that the lower level of CNS CTL exhaustion stems from the long time period required by clone 13 to inundate the brain parenchyma with antigen. However, it is important to note that despite this better preservation of function, CTL still failed to prevent clone 13 from establishing widespread persistence throughout the brain parenchyma. In fact, the duration of clone 13 persistence in the CNS was considerably longer than that observed in peripheral tissues containing heavily exhausted T cells. Therefore, the degree of functional exhaustion cannot be used to explain the pattern of persistence in the CNS following an intravenous clone 13 infection.

One of the most important features of the clone 13 model is the ability of dysfunctional antiviral CTL to regain their cytokine-producing abilities starting around day 60 p.i. This progresses to a state of complete functional recovery in all tissues examined by day 90 p.i. (Fig. 5). During this "reanimation phase" clone 13 is purged from most of the periphery [19,20], and we noted a marked elevation in the number of CTL in the CNS. Associated with CTL reactivation was a dramatic shift in the immune repertoire found in the CNS, but not the periphery (Fig. 6). Most notably, a substantial increase in CD4^+ ^T cells (which included the dominant I-A^b^GP_61–80 _specific response) and B cells were observed in the CNS. B cells and antiviral antibodies have been implicated in the control of certain strains of LCMV [58,59] as well as the CNS-tropic mouse hepatitis virus (JHMV) [60,61]. In addition, studies have shown that passive administration of anti-LCMV antibodies (IgG2a isotype) can partially protect mice from the fatal choriomeningitis induced by LCMV [62]. Collectively, these data suggest that diversification of the adaptive immune repertoire, which includes the mobilization of CD4^+ ^T cells and B cells, is responsible for the eventual clearance of clone 13 from the CNS, and, quite possibly, the periphery. Immune cell depletion studies are currently underway to evaluate this hypothesis.

In conclusion, our studies provide the first comprehensive profile of clone 13 replication and the responding adaptive immune response in the highly specialized CNS. These studies provide a framework for understanding how a host successfully purges a persistent infection from the CNS after immune defenses are hampered for several months by an immunosuppressive milieu. Given that this scenario is the desired outcome in persistently infected humans, we propose that a detailed examination of the natural instruction provided in the clone 13 system should reveal novel therapeutic strategies to eradicate persistent viruses. Finally, it is important to note that the clone 13 model, which is widely used to study peripheral modes of immunosuppression, also provides an excellent paradigm to examine viral-immune interactions within the CNS. The reanimation phase observed late in this model should be of particular use to those intending to examine the impact of immune repertoire diversification on CNS viral persistence.
